# Impact of climate change on shumbrite small scale irrigation project, South Gojjam subbasin, Ethiopia

**DOI:** 10.1016/j.heliyon.2023.e16352

**Published:** 2023-05-18

**Authors:** Tadege A. Worku, Tadele F. Aman, Melsew A. Wubneh, Temesgen M. Manderso

**Affiliations:** a Department of Hydraulic and Water Resources Engineering Debre Tabor University, Debre Tabor, Ethiopia; b Department of Hydraulic and Water Resources Engineering University of Gondar, Gondar, Ethiopia

**Keywords:** CORDEX, Climate change, CROPWAT, GCM, Shumbrite catchment

## Abstract

Climate change has the potential to affect climate parameters like rainfall and temperature which lead to a change in the irrigation water requirement of the irrigation system. As irrigation water requirement is highly dependent on precipitation and potential evapotranspiration, climate change impact studies are necessary. Therefore, this study aims to assess the impact of climate change on the irrigation water requirement of the Shumbrite irrigation project. For this study, climate variables of precipitation and temperature were generated from CORDEX-Africa simulations downscaled from MPI Global Circulation Model (GCM) under three emission scenarios (RCP2.6, RCP4.5, and RCP8.5). The climate data covers from 1981 to 2005 for the baseline period and 2021–2045 for the future period for all scenarios. Future precipitation shows a decrease for all scenarios with a maximum decrease under RCP2.6 (4.2%) and temperature show an increase in the future as compared to the baseline period. The reference evapotranspiration and Irrigation Water Requirements (IWR) were calculated by using CROPWAT 8.0 software. Results showed that the mean annual reference evapotranspiration is expected to increase in the future by 2.7%, 2.6%, and 3.3% for RCP2.6, RCP4.5, and RCP8.5 respectively as compared to the baseline period. Mean annual irrigation water requirement shows an increase of 2.58%, 0.74%, and 8.4% for the future under RCP2.6, RCP4.5, and RCP8.5 respectively. The Crop Water Requirement (CWR) also increases for the future period under all RCP scenarios, with maximum CWR for tomato, potato, and pepper crops. To ensure the sustainability of the project, crops with high irrigation water requirements should be replaced by other crops with low water requirements.

## 1 Introduction

Climate change is defined as the state of climate identified by changes in the mean and/or variability of its properties for long periods, whether due to natural activity or as a result of human activity. For example, influences such as changes in solar radiation and volcanism, occur naturally and contribute to the natural variability of the climate system [1]. Temperature fluctuations and changes in precipitation trends influence the components of the hydrological cycle and the availability of water supplies in general. The extent and seasonality of precipitation are altered by global temperature and atmospheric circulation rises and may contribute to an overall increase in the rate of evaporation and change in precipitation patterns [2].

The understanding of the effects of climate change as a result of human actions and influences is becoming accelerated from time to time. Climate change and its instability have many impacts on the hydrological cycle and, hence, on the world’s water supply systems. This reality has been confirmed by the Intergovernmental Panel on Climate Change, and greenhouse gases have played a major role in global and regional climate change [3]. The introduction of these gases into the atmosphere has disrupted the atmosphere's natural composition [4].

Due to the strong relationship between the hydrologic cycle and the climate system, any change in the climate could affect climate parameters such as precipitation, temperature, runoff, stream flow, and groundwater level, and this in turn leads to the change in crop water requirement of the agriculture [5]. Because the Changes in rainfall patterns are one of the most prominent consequences of climate change in the world. Crop water requirement is principally dependent on the amount of precipitation and the potential evapotranspiration [6].

Even if climate change has many impacts globally, the largest impact expected is on the agricultural sector. In developing countries like African countries, climate change can highly affect the sustainability of the agriculture system and people who depend on local food production will therefore be challenged. The severity of climate change impact in Africa increases in arid and semi-arid areas where water resource is sensitive to climate variability [7].

When climate changes, water demand for crop cultivation increases, Therefore, human beings should design strategies to adapt to this situation [8]. Furthermore, to manage agricultural water, an understanding of the impact of climate change on agricultural water consumption is very important. From this point of view, climate change results in a negative effect on evapotranspiration and Crop Water Requirements (CWR) [9]. The CWR value can be used as a measure of water availability and agricultural water requirements. So, it shows either water abundance or scarcity. Therefore, reasonable changes in CWR in the future are essential for water resource planning [6].

Among developing countries in Africa, Ethiopia is taken as one of the most vulnerable countries to the variability of climate [10]. This again results in a significant impact on the economy of the country. Because the agricultural sector highly contributes to the GDP of the country. Climate change has an impact on the quality, quantity, and availability of irrigation water. Therefore, the assessment of climate change's impact on irrigation as a general has to be done for setting mitigation measures.

Different studies have been conducted on the impact of climate change on irrigation water demand in Ethiopia. For example [11], assessed the dual impact of climate change on irrigation water demand and reservoir performance: a case study of the Koga irrigation scheme, in Ethiopia, and [7] evaluated the impact of climate change on maize production in Ethiopia. These studies have been conducted on large-scale irrigation levels (even at the national level). But it is necessary to understand the potential impact of climate change on small-scale irrigation schemes for planning and designing appropriate adaptation strategies. Therefore, this study has attempted to fill the gap by assessing the impact of future climate change on Crop Water Requirements (CWRs) as well as on the overall scheme's water requirement.

To analyze the potential impact of climate change, climate variables including rainfall and temperature were downscaled from CORDEX-Africa, MPI Global Circulation Model (GCM) under three emission scenarios (i.e., RCP2.6, RCP4.5, and RCP8.5) for baseline and future periods. CROPWAT model was used to simulate climate change impact on reference evapotranspiration and irrigation water requirement of the scheme.

## 2 Materials and methods

### 2.1 Description of the study area

**Fig. 1:**
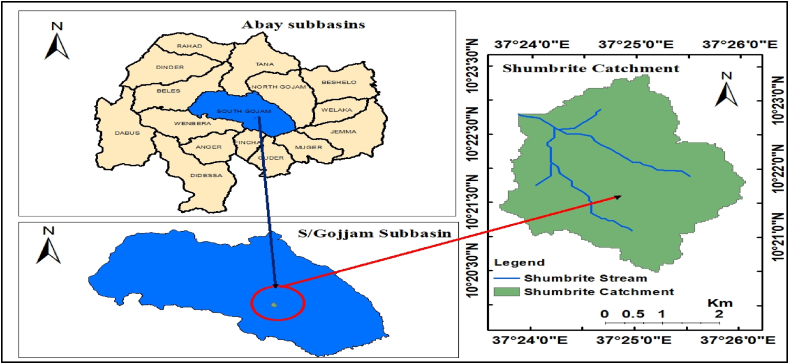
Location of Shumbrite catchment, South Gojjam subbasin.

This study is carried out on the command area of the Shumbrite reservoir located in the Abay basin, Ethiopia (Fig. 1). Shumbrite reservoir is a small-scale irrigation project constructed on the Shumbrite river which is the left tributary of Jedeb river. The catchment is mainly found in South Gojjam Subbasin with UTM coordinate of 11473000 m North and 324999.95 m East, and an area of 15 km^2^ at the dam site. The climate of the study area falls under the traditional agro-climate Zone of Woina Dega (sub-tropical) having an elevation of 1500–2500 m. The study area gains an annual rainfall of 1491.6 mm. The mean maximum and minimum temperatures are 30.1 °C and 9.7 °C respectively. The soil group dominated in the area is Vertisol and land use cover in the area is cultivated land in a high percentage and grassland in a small portion. The net irrigable command area to be cultivated using the Shumbrite reservoir was determined to be 270ha (Shumbrite Irrigation Design Report, 2015).

### 2.2 Data used

In this study, appropriate data has been identified and collected before the research work. The data used in this study include GCM simulated climate data, gauged climate data, and data from laboratory work. The primary data is mainly obtained from laboratory work. The secondary data such as precipitation and temperature were collected from Debre Elias Meteorological station for bias correction of GCM output rainfall and temperature data. Other data such as wind speed and sunshine hour were collected from Debre Markos station to develop an equation for relative humidity.

#### 2.2.1 Primary data

In addition to secondary data, primary data related to the project has been collected. The primary data collected for the Shumbrite irrigation project were soil samples for the determination of different soil parameters. As the command area of the project is not large enough, it was only divided into two plots (i.e. upper and lower) and a soil sample was taken from each plot. Based on the flow direction, the upper plot is around the entrance of the main canal and the Lower plot is around the end of the command area. The main soil parameters determined for this study include soil texture, bulk density, field capacity, permanent wilting point, and water availability. The soil parameters are determined to estimate both the baseline and future period crop water requirement and overall irrigation water requirement. Even if the parameters may change over time. It is difficult to project in this scope of the study.

##### 2.2.1.1 Soil properties

Soil Moisture Content

The soil sample was collected to determine the moisture of the soil from the two plots of an irrigated area with a depth interval of 30 cm and a maximum depth of up to 90 cm (i.e., 0-30.30-60 and 60-90). The samples were dried in an oven for 24 h at a temperature of 105 °C. After drying the sample, the soil and container are weighted together, and hence the weight of water is determined following pre- and post-readings (see equation (1)).

Soil moisture content by weight ($\theta_{w}$) can be estimated as follows.

(1) $$\theta_{w}=\frac{W_{w}-W_{d}}{W_{d}}*100$$

Where W_w_ is the wetted weight of soil (g), W_d_ is the dry weight of soil (g) and $\theta_{w}$ is soil moisture content based on dry weight (%)

The soil moisture content can be converted to volumetric water content ($\theta_{v}$) by multiplying with $\frac{bulk\;density\;of\;soil\left(\rho_{b}\right)}{bulk\;density\;of\;water}$, as in equation (2), however, the bulk density of water is close to unity. The equation becomes as follows.

(2) $$\theta_{v}=\rho_{b}*\theta_{w}$$

Soil textural class

To determine soil texture, soil samples at specified depths (i.e., 0–30 cm, 30–60 cm, and 60–90 cm) were taken at each plot (i.e Upper and Lower), and then soil particle size composition was calculated in the laboratory. Based on the percentage of composition, the textural class was determined using USDA SCS textural triangle. As shown in Table 1, silt loam soil is the dominant soil in both upper and lower plots.

Bulk density

**Table 1:** Soil properties of the selected fields for the Shumbrite irrigation scheme.

soil properties		Upper	Sampling Location		Lower	
	
Soil depth (cm)	0–30	30–60	60–90	0–30	30–60	60–90
%Sand	25.36	13.12	20.06	20.36	12.12	25.06
%Silt	48.64	61.38	52.44	58.64	61.38	55.44
%Clay	29.0	25.5	26.5	25	25.5	20.5
Textural class	Loam	Silt loam	Silt Loam	Silt loam	Silt loam	Silt loam
Bulk density (g/cm3)	1.12	1.07	0.98	1.0	1.07	1.11
FC(%vol)	29.92	30.24	30.79	25.53	28.49	32.65
PWP(%vol)	13.97	12.83	14.53	13.76	14.97	14.27
TAW (mm/m)	159.5	174.1	162.6	117.1	135.2	183.8

The bulk density of the soil was determined by drying the sample in an oven at 105 °C and weighing the soil and container. Finally, the dry weight of the soil is divided by its volume to determine dry bulk density. As shown in Table 1, The bulk density varies as high as 1.12 g/cm^3^ and a minimum of 0.98 g/cm^3^. According to Ref. [12], bulk density for silty soil should be less than 1.4 gm/cm^3^ for better plant growth. The soil class in this study is almost silty soil with good bulk density and hence suitable for plant growth.

Field capacity, Permanent wilting point, and Total available water

The moisture content at field capacity and wilting point were determined using samples from two sampling pits at depth intervals of 30 cm. To determine total available water (TAW), soil Field Capacity (FC), and Permanent Wilting Point (PWP) were first determined. The FC and PWP were determined through a pressure gauge apparatus of 0.33 bar and 15 bar respectively in the laboratory.

According to Ref. [13], TAW was computed as follows (equation (3)).

(3) TAW=(FC−PWP)

Where TAW is the total available water, FC is field capacity and PWP is the permanent wilting point. All parameters in the equation are in percentage by volume. The total available water can be best expressed in depth (mm/m) as in equation (4) below.

(4) $$TAW=\frac{\left(FC-PWP\right)}{100}*\frac{1000mm}{m}=10*\left(FC-PWP\right)$$

As it is shown in Table 1, the soil moisture content at field capacity (FC) varied from a minimum of 25.53% to a maximum of 32.65% by volume. The soil moisture content at the permanent wilting point (PWP) varied from 12.83% to 14.97% by volume. The value of the total available water (TAW) also varied from 117.1 mm to 183.8 mm.

### 2.3 Methodology

The methodology of this study mainly includes four parts, these are (1) Future climate data generated using the MPI climate model, (2) Bias correction of simulated climate data, (3) Calculation of ET_O_ using CROPWAT, (4) Calculation of irrigation water requirement.

#### 2.3.1 Climate data extraction

The climate change scenarios data from Coupled Model Intercomparison Project phase 5(CMIP5) RCM(RCA4) ensemble output of CORDEX-Africa under Representative Concentration Pathways (RCP2.6, RCP4.5and RCP8.5) were extracted from MPI climate model to be used as input to CROPWAT model. The downscaled climate data includes precipitation, and maximum and minimum temperature for the base period (1981–2005) and future period (2021–2045).

#### 2.3.2 Bias correction

RCM models offer data with a high degree of resolution. One of its limitations is that systematic errors occur during the initial data rectification. Bias may result from theoretical and practical constraints; this bias should be removed utilizing correction methods. In this study, the temperature was corrected using the Quantile Mapping (QM) method, while rainfall was corrected using the Power Transformation method.

**Precipitation bias correction**: Finer level of local climate replication is possible with RCMs. The model output should be post-processed because findings from RCM cannot be used as direct input data for hydrological models due to systematic flaws. [14]. Precipitation is generally erratic in its spatial distribution and highly nonlinear. A nonlinear method for adjusting both the mean and variance of a precipitation data set is the Power Transformation (PT) method. In this study, precipitation is typically corrected by the Power Transformation method. This correction approach compares daily observed precipitation at Debre Elias station (1981–2005) with the RCM's nearest grid point, treating the grid points as a single station on the watershed. The power transformation method can be expressed by the following equation (equation (5)).

(5) P* = a *P^b^

Where P is uncorrected precipitation P* is corrected simulated precipitation, a and b are parameters calculated by equating the coefficient of variation (CV) of corrected P^b^ and CV of observed precipitation at a similar correction period.

**Temperature bias correction (T**_**min**_**and T**_**max**_**):** The daily temperature was corrected by using one of the best bias correction methods known as the Quantile Mapping (*QM*) method by using a Climate Data Bias Corrector (*CDBC*) tool. The *CDBC* tool corrects both historical and future time climate data (i.e., Pr and Temp). The correction for the historical period is done by adding observed and historical temperature data under a software menu called ’for historical Data’ and for the future time, the three temperature time series data (i.e., observed, historical, and future) are added to a menu called ‘for Future Data’ and finally the corrected data is obtained in the specified output folder [15].

#### 2.3.3 Representative concentration pathway (RCP)

RCPs are time- and space-dependent trajectories of greenhouse gas and pollutant concentrations and emissions resulting from human activities, such as land use change. RCPs give a quantitative estimate of climate change pollutant concentrations in the atmosphere through time, as well as their radiative forcing in 2100. Researchers from different fields involved in climate research collaborated to develop RCPs [16]. RCP 2.6, RCP4.5, RCP6, and RCP8.5 are the four RCPs that have been developed. The radiative forcing target levels of 2.6, 4.5, 6, and 8.5 W/m^2^ at the end of the twenty-first century were used to name the RCPs [17]. The radiative forcing is calculated depending on the forcing of GHGs. These four distinct pathways each define a unique emissions trajectory and radiative forcing. Based on this, RCP2.6 is a low-emission scenario, both RCP4.5 and RCP6 are medium-emission scenarios and RCP8.5 is a high-emission scenario. For this study, only three RCPs such as RCP2.6, RCP4.5, and RCP8.5 are used to assess the impact of climate change on the irrigation requirement.

#### 2.3.4 ET_o_ calculation

In this study, monthly potential evapotranspiration (PET) was estimated using CROPWAT 8.0 software which uses the Penman-Monteith method. The penman monteith method is the commonly used method to determine PET. To calculate potential evapotranspiration for the future period using the Penman-Monteith method mean monthly data of temperature, humidity, wind speed, and sunshine hour are required. However, the existing data were the only downscaled precipitation and minimum and maximum temperature. But, according to Ref. [5], it is assumed that wind speed would not significantly change in the future time. For this study sunshine hour and wind speed are assumed to be constant for the future. Monthly relative humidity (RH) is supposed to be a function of temperature (Tmax Tmin), sunshine hour (Sun), and rainfall (Rain) as shown below (equation (6)).

(6) RH=f(Tmin,Tmax,Sun,Rain)

Using multivariable linear regression, a formula was developed to calculate relative humidity (RH) for the base period and future period (equation (7)). The correlation coefficient of the regression function is 0.98 which is acceptable.

(7) RH=0.0045Rain+4.596Tmin−4.92Tmax−1.4769Sun+145

Finally, potential evapotranspiration is calculated using CROPWAT-8 software for the base period (1981–2005) and future time (2021–2045).

#### 2.3.5 Calculation of irrigation water requirement

The calculation of crop water requirements and irrigation requirements is carried out using inputs of climatic, crop, and soil data. The climatic data (precipitation and temperature) was obtained from GCM output. Other climatic data was taken from the gauge and assumed to be constant as stated in section 2.3.4, and relative humidity was computed using equation (7) for both baseline and future periods. The crop data were obtained from the design report and field visit and soil data was from the laboratory and used for future irrigation water demand projection.

To estimate crop water requirements and irrigation water requirements CROPWAT requires:

***Reference Crop Evapotranspiration (ETo):*** It is calculated using the Penman-Monteith equation based on decade/monthly climatic data; minimum and maximum air temperature, relative humidity, sunshine duration, and wind speed using CROPWAT software. The temperature data was from GCM output, sunshine hour and wind speed are from the gauge,

***Rainfall****:* Rainfall was extracted from the MPI model under the three RCPs for baseline and future periods and used for the estimation of effective rainfall. A method of ‘dependable rain’ was used to determine effective rainfall as stated in equations (8), (9)) below.

(8) Effective Rainfall = (0.6*total rainfall) −10, (total RF < 70 mm)

(9) Effective Rainfall = (0.8*total rainfall)-24, (total RF > 70 mm) .

***Cropping pattern****:* A cropping pattern made up of the area planted, the planting date, and crop coefficient data files (such as Kc, stage days, root depth, and depletion %). A set of typical crop coefficient data are obtained from FAO Irrigation and drainage papers No.24 and No.56. The main crops, cropping patterns, and the percentage area coverage are given in Table 2 below.

**Table 2:** Cropping pattern of shumbrite small-scale irrigation project.

S. No	Cropping Pattern	Area (ha)	%Area	planting date	Harvesting date
1	Maize	15	5.6	1- Dec	10- Apr
2	Wheat	1	0.4	1-Dec	10-Apr
3	Onion	25	9.3	1-Feb	22-May
4	Tomato	30	11.1	15-Dec	25-Mar
5	Cabbage	10	3.7	15-Dec	25-Mar
6	Potato	150	55.6	1-Jan	1-May
7	Carrot	15	5.6	1-Dec	21-Mar
8	Pepper	3	1.1	20-Dec	9-Apr
9	Beetroot	21	7.8	1-Dec	21-Mar
	Total	270	100		

***Soil data:*** For that particular site, laboratory work yielded the total available moisture depletion (percent of total available moisture), and other information, such as the moisture depletion fraction, was taken from FAO papers (i.e., For cultivated soil moisture varies from 20% to 70%). A 45% depletion fraction was used for this study.

##### 2.3.5.1 Calculation method

For calculating crop evapotranspiration, reference crop evapotranspiration (from CROPWAT) and crop coefficient were used based on equation (10) below.

(10) $$ET_{C}=K_{C}*ET_{o}$$

Where ETc is crop evapotranspiration is crop coefficient and ET_o_ is reference crop evapotranspiration.

For a given crop i, and cropping period j, the model calculates the monthly crop water requirements using equation (11) below.

(11) $$CWRj=\sum_{i=1}^{n}\left(K_{cit}*{ET}_{o}-P_{eff}\right)$$

Where, Kcit is the crop coefficient of (a given crop i, during the stage t, n is the total number of crops in that period).

Then, the net irrigation water requirement (NIWR) in a specific scheme for a given period is estimated by multiplying individual crops' water requirements and irrigation area in a specific scheme for a given period as shown below (equation (12)).

(12) NIWRj=CWRj*Sj

Where, Sj is the total area in period j, for a specific scheme, NIWRj is the net irrigation water requirement for period j, and CWRj is the crop water requirement in period j.

To account for losses of water incurred during conveyance and application to the field, an efficiency factor should be included when calculating the irrigation water requirements for a scheme. This efficiency factor results in the gross irrigation water requirements (GIWR) per period. The GIWR can be calculated using Equation (13) below.

(13) $$GIWR=\frac{NIWR}{E_{P}}$$

Where GIWR is gross irrigation water requirement, NIWR is net irrigation water requirement and E_P_ is project efficiency. For this specific study, the overall efficiency of 60% is taken from the design report and considered constant for the future period.

## 3 Result and discussions

### 3.1 Bias correction

Because there are some uncertainties in the raw GCM output data, it is required to correct the data using the proper technique and compare it to observed or gauge data to ensure accuracy. In this study, both temperature and precipitation data has corrected and a correlation was made with observed data to see the agreement between them.

**Fig. 2:**
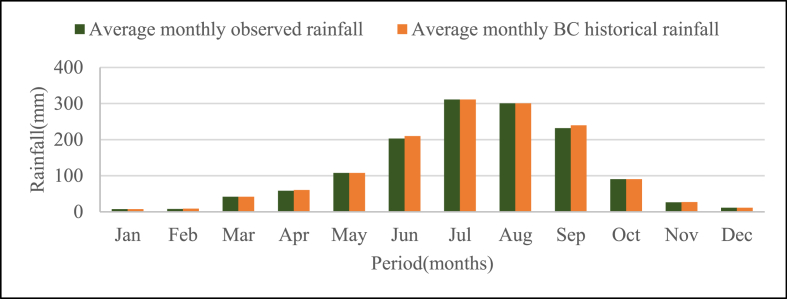
Observed and Bias corrected RCP historical mean monthly Rainfall for the base period (1981–2005).

The result of monthly average bias corrected RCP rainfall data and observed rainfall within the base period shows a good agreement. The RCP rainfall at the base period has a correlation coefficient of 0.99 with the observed data as shown in Fig. 2.

**Fig. 3:**
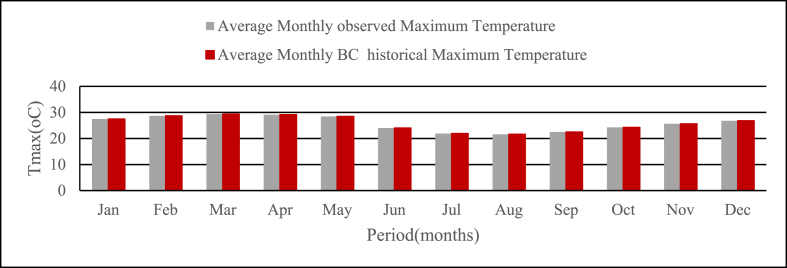
Average monthly Observed and Bias corrected historical Tmax at base period for study area from (1981–2005).

**Fig. 4:**
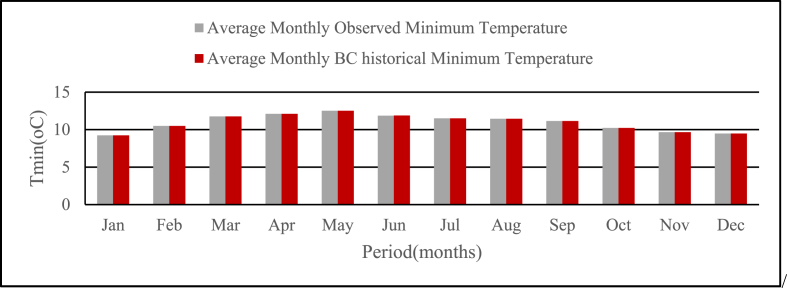
Average monthly Observed and Bias corrected historical Tmin at base period for the study area from (1981–2005).

Similarly, the bias-corrected maximum and minimum RCP historical temperature at the base period has a correlation coefficient of 0.98 with the observed temperature as shown in Fig. 3, Fig. 4 below.

### 3.2 Future climate variables

#### 3.2.1 Precipitation anomalies

**Fig. 5:**
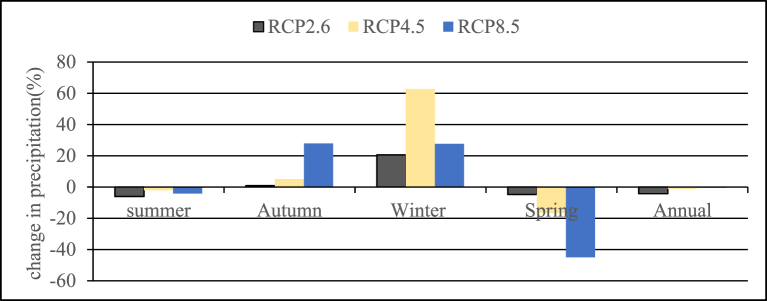
Percentage change in Bias corrected mean annual and seasonal future precipitation.

The mean annual precipitation will decrease for all scenarios for future time series compared to the base period with a maximum decrease in RCP2.6 (4.2%). In comparison to the baseline period, one season's predicted precipitation distribution under RCP scenarios shows a rise, while another season shows a drop. Accordingly, for RCP2.6, RCP4.5, and RCP8.5 scenarios the seasonal change of precipitation compared to the base period shows a decrease in the Summer and Spring seasons with a maximum decrement of (6.06%) and (44.97%) respectively and an increase in Autumn and Winter seasons with a maximum increment of (27.87%) and (62.76%) respectively. The change in seasonal and annual rainfall over the Shumbrite River catchment during the future period of 2021–2045 is presented in Fig. 5 below.

#### 3.2.2 Temperature anomalies

**Fig. 6:**
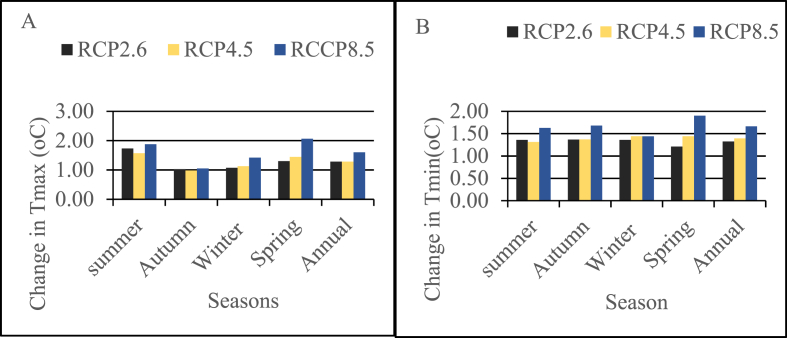
Change in Bias corrected mean annual and seasonal temperatures with comparison to the baseline period (A) maximum temperature (B) minimum temperature.

In comparison to the baseline period, the distribution of the mean annual and seasonal temperatures (both minimum and maximum) predicted by the GCM shows an increase in the future. The mean annual maximum temperature increases by 1.28 °C under RCP2.6 and RCP4.5 and by 1.6 °C under RCP8.5 (Fig. 6A). The annual minimum temperature also increases by 1.32 °C and 1.39 °C under RCP2.6 and RCP respectively and by 1.66 °C under RCP8.5 (Fig. 6B). Similarly, for the seasonal distribution, both simulated maximum and minimum temperatures under all RCPs show an increase in all seasons but the maximum increase is at RCP8.5. Generally, the summer season shows a maximum increment in mean seasonal maximum temperature, and Autumn shows a minimum increment in mean seasonal maximum temperature under all RCPs. From the result, it can be deduced that the mean seasonal maximum temperature under all scenarios will tend to greater warming in the summer season.

#### 3.2.3 Reference crop evapotranspiration

**Fig. 7:**
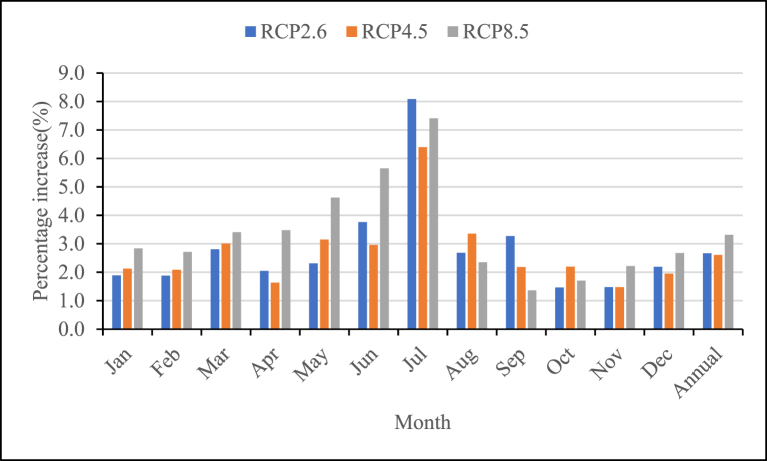
Monthly change of future reference crop evapotranspiration as compared to the baseline period.

The reference crop evapotranspiration was calculated using Penman’s method based on the climate data simulated from the GCM model. It mainly depends on the temperature trend. As in section 3.2.2, temperature shows an increase under all scenarios. Due to the increase in temperature, the reference evapotranspiration tends to increase in the future period for all scenarios (Fig. 7).

The mean annual reference Evapotranspiration shows an increase of 2.7%,2.6%, and 3.3% for RCP2.6, RCP4.5, and RCP8.5 respectively as compared to the baseline period (Fig. 7). As presented in Table 3, the monthly future reference evapotranspiration has a maximum value in March, April, and May and a minimum value in July and August for all RCP scenarios.

**Table 3:** Reference crop Evapotranspiration (mm).

Month	Jan	Feb	Mar	Apr	May	Jun	Jul	Aug	Sep	Oct	Nov	Dec
Baseline	131	134	155	147	148	112	92.1	92	110	127	128	127
RCP2.6	134	137	159	150	151	116	100	95	114	129	124	130
RCP4.5	134	137	159	149	152	115	98	96	113	130	124	130
RCP8.5	135	138	160	152	154	118	99	95	112	129	125	131

### 3.3 Future irrigation water requirement

The crop water requirement was calculated based on GCM simulated climate parameters and shows an increase for all crops under all RCP scenarios. Reference crop evapotranspiration and crop coefficients were used to calculate crop evapotranspiration. The increase in future crop evapotranspiration will increase the crop water requirement. As shown in Table 4, the crops with the maximum water requirement are tomato, potato, and pepper. The crop water requirement increases up to 2.9%,2.6%,3.1% for RCP2.6,3.6%,3.3%,6.4% for RCP4.5, and 5.2%,5.7%,8.4% for RCP8.5 For tomato, potato, and pepper respectively.

**Table 4:** Comparison of crop water requirement between baseline and future period.

	Crop Water Requirement (mm)			
Crops	Baseline	RCP2.6	RCP4.5	RCP8.5
Maize	477	492.8	493	497.8
Wheat	455.4	470.9	471.4	476.4
Cabbage	424.4	440.2	440.7	443.1
Tomato	573.9	590.4	594.6	603.7
Potato	554.5	568.7	572.8	585.9
Pepper	490.5	505.6	522	531.7
Onion	412.1	423.4	426	438
Carrot	387.2	395.7	396	398.9
Beet root	387.2	395.7	396	398.9

The irrigation water requirement of the scheme was calculated for the baseline period and future period under the three RCP scenarios (RCP2.6, RCP4.5, and RCP8.5). In addition to crop evapotranspiration, effective rainfall was calculated using the soil conservation service (SCS) method to calculate irrigation water requirements. The current net irrigable command is 270ha and is assumed to be constant for the future period. The mean annual water demand volume shows an increase of 2.58%,0.74%, and 8.4% under RCP2.6, RCP4.5, and RCP8.5 respectively as compared to the base period. A similar study was conducted by Ref. [11]on the Koga irrigation project Using RCP4.5 and RCP8.5. The authors have affirmed that irrigation water is projected to increase in the future under both scenarios. This study has also confirmed similar results. Generally, climate change will have a far-reaching effect on evapotranspiration and irrigation water requirement of small-scale irrigation projects. The comparison of monthly irrigation water requirements is presented in Table 5 below.

**Table 5:** Comparison of irrigation water requirement between baseline and future period.

	Irrigation Water Requirement (mm)			
Month	Baseline	RCP2.6	RCP4.5	RCP8.5
Jan	129.9	132.7	132.7	133.6
Feb	214.3	219.1	219.5	220.8
Mar	244.6	259.4	259.8	261.4
Apr	187.0	187.5	188.7	200.8
May	38.7	39.8	41.4	42.9
Jun	0	0	0	0
Jul	0	0	0	0
Aug	0	0	0	0
Sep	0	0	0	0
Oct	0	0	0	0
Nov	0	0	0	0
Dec	36.4	36.6	37.0	37.4

## 4 Conclusion

This paper assesses the impact of climate change on the irrigation water requirement of the Shumbrite small-scale irrigation project. To determine irrigation water requirement, climate data including precipitation, and maximum and minimum temperature were downscaled from the MPI model under the three scenarios (i.e., RCP2.6, RCP4.5, and RCP8.5). The analysis of climate variables from the MPI climate model indicates that the mean annual precipitation shows a decrease under all scenarios for future time series compared to the baseline period with a maximum decrease of 4.2% under RCP2.6. The mean annual maximum and minimum temperature shows an increase in the future period as compared to the baseline period under all RCP scenarios. The mean annual maximum temperature changes from 1.28 °C to 1.6 °C having a maximum increase for RCP8.5. The annual minimum temperature also changes from +1.32 °C to +1.66 °C and the maximum increase is obtained for RCP8.5. Based on the downscaled climate data, reference evapotranspiration was estimated under all RCPs. As a result of an increase in temperature, future reference evapotranspiration tends to increase for all RCP scenarios with a maximum increment under RCP8.5 (3.3%). The analysis of the irrigation water requirement result showed that the mean annual irrigation water requirement increases for all RCPs with a maximum increase (8.4%) under RCP8.5 as compared to the baseline period. The maximum crop water requirement was observed for tomato, potato, and pepper crops in the future period. Despite the increase in temperature and decrease in precipitation, irrigation water requirement increases for the future period. Hence, to ensure the sustainability of the project, crops with high irrigation water requirements should be replaced by other crops with low water requirements. It is helpful to cultivate crops such as wheat, cabbage, onion, and barley in the area. Because these crops have relatively low water requirements. The limitation of this study can be outlined as it uses a single GCM, so future studies should incorporate more GCMs to assess climate change impact. In addition to this, the limitation is that the study assesses the impact of climate for climate variables of precipitation and temperature the other climate variables such as wind speed and sunshine hour were assumed to be constant in the future. Wind speed was assumed to be constant based on [5]. Another limitation of this study is that the GCM output data is downscaled based on Coupled Model Intercomparison Project Phase 5(CMPI5). Because the newly developed project CMPI6 was not available during this study. So, it is recommended to use CMPI6 in future studies.

## Author contribution statement

Tadege A. Worku: Conceived and designed the experiments; Analyzed and interpreted the data; Wrote the paper.

Tadele F. Aman; Melsew A. Wubneh; Temesgen M. Manderso: Performed the experiments; Contributed reagents, materials, analysis tools or data.

## Funding statement

No funding source for this research.

## Data availability statement

Data included in article/supp. Material/referenced in article.

## Declaration of competing interest

The authors declare that they have no known competing financial interests or personal relationships that could have appeared to influence the work reported in this paper.
